# Turning over New Leaves

**Published:** 1888-05-26

**Authors:** 


### TURNING OVER NEW LEAVES.

[Books for Review should be sent to The Editor, The Lodge, Porchester Square, W.]

### A New Irish Novel.*

IT is so long since the author of "Hogan, M.P.," the creator of those three delightful beggar children, Flitters, Tatters, and the Counsellor, gave us a serious essay in fiction, that readers of "Ismay's Children" will take it up with some curiosity, to see if the old brilliancy of writing and close observation of the external characteristics of people and places are still present in the writer's work. They are; and there is also the old defect—that the plot is the least interesting part of the book, and this in spite of the plot of "Ismay's Children" being a good one. No one is really much interested in the claim of the young Mauleverers to the Barrettstown estate, held in possession by their distant cousin, Tighe O'Malley, because their semi-paralysed aunt, Juliet D'Arcy, has forgotten the name of the place where their dead parents were married. It is constantly alluded to, yet it lays little hold of the reader's imagination, and even the fine incident of Godfrey Mauleverer going, at the risk of his life, to warn the usurping O'Malley of an approaching attack of the Fenians, rouses less sympathy than it ought. As a matter of fact, Mrs. Hartley's strong point is not story-telling, but the depicting of characteristic scenes of life in Ireland, and on however strong a thread of plot these may be strung, it never becomes an integral part of the work. The vividness and accuracy of these scenes everyone who has travelled in south-western Ireland will recognise, while those who have not may learn from these pages many of the features of a nation which most English people seem ready to describe with the magnificent impartiality of ignorance. The circumstances attendant on land tenure in the sister island are very clearly set forth, and will surprise many who have looked in vain through more pretentious works for any clear statement of a problem which it seems impossible to solve. The farmers, the shopkeepers, the peasants, all want land; it is the only investment they care for. To obtain it they will not only give twice as much rent as the ground is worth, but will bid over each other from twenty to fifty per cent. above the original offer. It would be asking too much from human nature to expect a landlord to refuse the high terms offered—indeed, thrust upon him; and thence arise evictions and rack-rents. The old tenant, whose family has perhaps been on the land for a hundred years, feels wronged when he is turned out to give place for a new-comer, who happens to have more money than he, and cherishes vengeful thoughts. Meanwhile the new tenant goes on cheerfully paying as rent more than he gets out of the land, so long as he has other funds at his back, or children in America, who will send their earnings home to help him. But he does nothing for the land, and as little as he can help for the buildings on the farm, because he knows that at the end of his lease he will have to pay a heavy "fine," or rather bribe, to the landlord in order to get it renewed, and every shilling he spends on improving his holding will have to be paid over again in this fine, if he does not want some outsider to step in, bid over him, and profit by his improvements. That this is a thoroughly wrong and false system is obvious, but landlords can hardly be regarded as actively to blame in taking the best terms they can get—market value is the accepted rate of all trade, and market value is the highest price that can be obtained by open competition. The remedy would seem to be the establishment of courts, whose members are practical agriculturists, to fix the proper rental of the land, and not permit tenants to pay a larger price, even when they propose to do so. Even such a court, however, supposing all its members to be capable and honest, must needs exist for a longer time than is granted to most Irish experiments before it could profit a people who are by hereditary habit short-sighted and incompetent farmers, and who resent as a personal wrong any interference with their accustomed ways. The marriage customs of the people are described in "Ismay's Children" with much frankness and humour—the business like way in which matches are made by parents and guardians with very little regard to the principals' feelings, or indeed to anything but the most mercenary considerations. The "fortune" is everything, the individual nothing—even though the said fortune be far from ample. Once, indeed, in County Kerry, we heard of an instance in which a young man's fancy—or his parents'—was led away from one girl to another by the fact that the second, though no richer in coin of the realm, would have an "oven pot" to her dowry more than her rival, and a pig or a brood of chickens will very often turn the scale. It is very rash to find fault with an

* "Ismay's Children." By the Author of "Hogan, M.P.," &c. London: Macmillan and Co.

author's English, because it seems to be generally conceded that, as Jeames de la Pluche said of spelling: "Every gentleman has his"—and still more, every lady has her—"own." Nevertheless we venture to join issue with Mrs. Hartley, who is in general a most accurate writer, on her use of the word "vicarious." We have always taken it to mean "by proxy"—as, for example, in the supreme instance in connexion with which the word is most frequently employed, the vicarious sufferings of our Lord are those which He endured instead of us; but in "Ismay's Children" it is habitually employed for "occasional"—*e.g.* "a sort of periodical fit of energy that had to be dissipated somehow or other, and which expended itself in fits of *vicarious* piano practice." This, however, is a small fault to find with a clever and interesting book.

### Medical Nursing.*

This is a simple and practical little book, and while it strictly marks out the nurse's position as subordinate to the doctor, gives clear and intelligent instruction in the duties she will have to perform, and the nature of the diseases she has to deal with. Dr. Anderson gives a number of useful hints on the making of poultices, fomentations, &c., besides a number of recipes for invalid cookery. The value of the work is increased by the glossary of medical terms at the end, and the list of questions appended to each chapter, so that every reader can examine herself on what she has learned from it. It might well find a place in every nurse's library.

### Public Health.†

"Public Health" is the new monthly journal of the Society of Medical Officers of Health, and is edited by Mr. Winter Blyth. As to its appearance it is small, neat, and presentable: as to its contents it is quite what might have been expected from its source and origin. It deals with preventive medicine and health preservation rather than with physic and cure. Its range of interest is broad and important, as well to the general public as to medical men; and we should be glad if the general public would read, mark, learn, and inwardly digest its contents. Bacteriology is, of course, well to the front, and is discussed with much clear simplicity and fullness of historical detail. The paper on this subject well illustrates Solomon's assertion that there is nothing new under the sun. The science of bacteriology is not quite as "old as the hills," but it is as old as the century, and very much older. It makes one unhappy to think that only a few of the initiated will be likely to read "*Public Health.*" And yet every master and matron would find both diversion, instruction, and profit in its pages.

### Prosperity or Pauperism?‡

The Earl of Meath, better known to the larger public as Lord Brabazon, has done well to publish in a cheap form a number of his contributions to periodical literature, on subjects which must always be of paramount interest to those who have intelligence enough to understand the difference between a man and a horse. It is a perpetual source of wonder to some persons how it can possibly happen that thousands of people, born and reared with every possible advantage of wealth, education, and religious training, should apparently have no more capacity for knowing the world they live in than a red Indian or a parish fool. Of the class to which the Earl of Meath himself belongs, how many will take the trouble to read his book? And of those few who do glance through its pages, what proportion will display any more interest in it than to pronounce it, with imbecile good nature, "a faddy production"? Of course, so far as those people themselves are concerned, we would not take the trouble even to utter a contemptuous word about them; but the problems with which the Earl of Meath deals are so great, so far-reaching, so difficult of solution, and productive of so much terrible misery to millions born and yet to be born, that it appals us to think of the stupid indifference of those who, by birth, education, and opportunity, should be in the very forefront of the battle in which the Earl of Meath is so ardent a leader. The book, to use an expression not unfamiliar to readers of seventeenth-century literature, "wrestles" with the great questions of work for the poor, wages for the workers, practical training for the young, education, physical exercise, fresh air, and, indeed, every other subject of urgent practical moment which forces itself upon the half-taught millions of our own and other countries. The millions are with us: they are indispensable to us: they are born, and they must be fed, taught, clothed, and housed in some way. It is not in them to form themselves into a perfectly-ordered state within the greater state in which they find themselves. They need leaders and guides, teachers and helpers; and any man of wealth, education, and leisure who strives to give them good leadership deserves credit and honour, at least, for his motives. We cannot do more than indicate, as we have already done, the nature of the contents of Lord Meath's book. It is an honest attempt to help where help is most urgently needed. It is written with vigour, intelligence, and a practical knowledge of the subjects treated. Its price is only five shillings; and we should be most pleased to hear that a hundred thousand copies had been sold within a month. It is better reading than will be found in a cartload of ordinary novels.

### A New Review.*

The "Universal Review" sounds a new and welcome note in magazine literature. Wider in scope than any existing review, it purposes to give its readers the thoughts of the best minds in many lands and in many languages—those of music and drawing, as well as words. With a poem by Lewis Morris and a novel of Daudet, an article in French on "Music in Balzac;" and a drawing by Sir F. Leighton, Mr. Quilter may well be thought to have attained what he desired. His own manly and unconventional criticism of the Academy, with its admirable illustrations, is not the least acceptable of the contents of the review, and will specially gladden those simple souls who have long cherished a dear, rebellious thought that a picture ought to be more than a glorified edition of the painter's palette, and that the great painters attained greatness by thinking simply and nobly of their subject as well as of their technique. Mrs. Lynn Linton's paper on Zola's "Idée Mère" is a little disappointing. It is incomprehensible to those who do not, and unsatisfactory to those who do know the writer, who has most conscientiously lavished pearls on swine; and who, scientifically desirous of showing the doctrine of heredity by means of fiction, has failed in his attempt through his having held too firmly the notion that "the evil that men do lives after them; the good lies oftimes buried with their bones." But Mrs. Lynn Linton does not seem to apprehend fully the essential flaw of Zola's artistic method—his avowed desire to seek out under all veils of custom and civilization "the brute within the man," forgetting that in man there is much besides the brute, many complicated and noble impulses which redeem his actions, and affect his children's characters. The general tendency of hereditary evolution is upwards, not downwards. Mrs. Crawford speaks of General Boulanger with the clearness and tone of thorough knowledge of the subject, and the other papers are of such a nature as to put the "Universal Review" in the front rank of our best monthly magazines.

---

I had rather feel compunction than understand the definition thereof.—*Thomas à Kempis.*

---

* "Medical Nursing." By J. Wallace Anderson, M.D. Glasgow: James Maclehose and Sons. Third edition: price 2s. 6d.

† W. Allen and Co., Ave Maria Lane, Paternoster Row.

‡ "Physical, Industrial, and Technical Training." Edited by the Earl of Meath. London: Longmans and Co.

* "The Universal Review," edited by Harry Quilter. (Swan Sonnenschein and Co.) Price, Half-a-crown.

